# Effect of an IT‐based social care intervention for dementia caregivers on unmet resource needs

**DOI:** 10.1002/alz70858_101635

**Published:** 2025-12-25

**Authors:** Soo Borson, Jennifer Makelarski, Kristen Wroblewski, Emily Abramsohn, Elbert S. Huang, SuYeon Lee, Katherine Thompson, Stacy Tessler Lindau

**Affiliations:** ^1^ Keck USC School of Medicine, Los Angeles, CA, USA; ^2^ BOLD Center on Early Detection of Dementia, New York, NY, USA; ^3^ University of Chicago, Chicago, IL, USA

## Abstract

Interventions for dementia caregivers should be scalable and available at the point of care. CommunityRx‐Dementia (CRxD) is an IT‐based intervention to improve caregiver outcomes by providing information about local community resources and connection to a resource navigator and online resource finder. We compared self‐reported unmet needs and resource knowledge among 343 caregivers enrolled in a randomized trial of CRxD vs usual care (UC). At baseline,1 and 3 months, caregivers’ reported their knowledge of and need for any of 14 resource types (e.g., respite care, end‐of‐life planning, food) and, at 12 months, they reported their use of acute care. For the outcomes of knowledge and needs, mixed‐effects regression models were fit with treatment arm, time, treatment arm by time interaction and baseline knowledge or needs as predictors. For acute care utilization outcomes, negative binomial regression models were fit with treatment group and baseline utilization as predictors. Incidence rate ratios (IRR) and corresponding 95% CIs were calculated. Participants (78% women, 81% non‐Hispanic Black, 49% aged 50‐64, 64% income ≥$50k/year) included both newer (22% 6 months‐2 years) and more experienced caregivers (44% > 5 years). At baseline, caregivers in both study arms reported an average of 4 unmet needs (87% ≥1, 65% ≥3, most often [61%] for caregiver education), and knew of an average of 6 resources. At 3 months, the number of known resources was greater in the CRxD than UC group (6.5 vs 5.2; β: 1.0, 95% CI 0.3, 1.7) and the number of unmet needs was lower (2.8 vs 3.6; β: ‐0.5, 95% CI ‐1.1, 0.0). Over 12 months, caregivers in CRxD had lower ED visits rates than UC participants (0.3 vs 0.5; IRR: 0.6, 95% CI 0.3, 0.9) but similar hospitalization rates (0.1 vs 0.2; IRR: 0.7, 95% CI 0.3, 1.2). This low‐intensity social care intervention for dementia caregivers may improve knowledge of available resources, decrease unmet needs, and reduce caregivers’ use of emergency care.

